# New Result of Analytic Functions Related to Hurwitz Zeta Function

**DOI:** 10.1155/2013/475643

**Published:** 2013-12-12

**Authors:** F. Ghanim, M. Darus

**Affiliations:** ^1^Department of Mathematics, College of Sciences, University of Sharjah, Sharjah, United Arab Emirates; ^2^School of Mathematical Sciences, Faculty of Science and Technology, Universiti Kebangsaan Malaysia, 43600 Bangi, Selangor Darul Ehsan, Malaysia

## Abstract

By using a linear operator, we obtain some new results for a normalized analytic function *f* defined by means of the Hadamard product of Hurwitz zeta function. A class related to this function will be introduced and the properties will be discussed.

## 1. Introduction

A meromorphic function is a single-valued function, that is, analytic in all but possibly a discrete subset of its domain, and at those singularities it must go to infinity like a polynomial (i.e., these exceptional points must be poles and not essential singularities). A simpler definition states that a meromorphic function *f*(*z*) is a function of the form

(1) $$\begin{array}{c} f\left(z\right)=\frac{g\left(z\right)}{h\left(z\right)}, \end{array}$$

where *g*(*z*) and *h*(*z*) are entire functions with *h*(*z*) ≠ 0 (see [1, page 64]). A meromorphic function therefore may only have finite-order, isolated poles and zeros and no essential singularities in its domain. A meromorphic function with an infinite number of poles is exemplified by csc(1/*z*) on the punctured disk U* = {*z* : 0 < |*z*| < 1}.

An equivalent definition of a meromorphic function is a complex analytic map to the Riemann sphere. For example, the Gamma function is meromorphic in the whole complex plane; see [1, 2].

In the present paper, we will derive some properties of univalent functions defined by means of the Hadamard product of Hurwitz Zeta function; a class related to this function will be introduced and the properties of the liner operator *L*_*a*_^*t*^(*α*, *β*)*f*(*z*) will be discussed.

## 2. Preliminaries

Let Σ denote the class of meromorphic functions *f*(*z*) normalized by

(2) $$\begin{array}{c} f\left(z\right)=\frac{1}{z}+\sum_{n=1}^{\infty }a_{n}z^{n}, \end{array}$$

which are analytic in the punctured unit disk U*. For 0 ≤ *β*, we denote by S*(*β*) and *k*(*β*) the subclasses of Σ consisting of all meromorphic functions which are, respectively, starlike of order *β* and convex of order *β* in U*.

For functions *f*_*j*_(*z*)  (*j* = 1; 2) defined by

(3) $$\begin{array}{c} f_{j}\left(z\right)=\frac{1}{z}+\sum_{n=1}^{\infty }a_{n,j}z^{n}, \end{array}$$

we denote the Hadamard product (or convolution) of *f*_1_(*z*) and *f*_2_(*z*) by

(4) $$\begin{array}{c} \left(f_{1}*f_{2}\right)=\frac{1}{z}+\sum_{n=1}^{\infty }a_{n,1}a_{n,2}z^{n}. \end{array}$$

Let us define the function $\tilde{\phi }(\alpha ,\beta ;z)$ by

(5) $$\begin{array}{c} \tilde{\phi }\left(\alpha ,\beta ;z\right)=\frac{1}{z}+\sum_{n=0}^{\infty }\frac{{\left(\alpha \right)}_{n+1}}{{\left(\beta \right)}_{n+1}}z^{n}, \end{array}$$

for *β* ≠ 0, −1, −2,…, and *α* ∈ *ℂ*/{0}, where (*λ*)*n* = *λ*(*λ* + 1)_*n*+1_ is the Pochhammer symbol. We note that

(6) $$\begin{array}{c} \tilde{\phi }\left(\alpha ,\beta ;z\right)=\frac{1}{z^{2}}F_{1}\left(1,\alpha ,\beta ;z\right), \end{array}$$

where

(7) $$\begin{array}{c} {\;}_{2}F_{1}\left(b,\alpha ,\beta ;z\right)=\sum_{n=0}^{\infty }\frac{{\left(b\right)}_{n}{\left(\alpha \right)}_{n}}{{\left(\beta \right)}_{n}}\frac{z^{n}}{n!} \end{array}$$

is the well-known Gaussian hypergeometric function.

We recall here a general Hurwitz-Lerch-Zeta function, which is defined in [3, 4] by the following series:

(8) $$\begin{array}{c} \Phi \left(z,t,a\right)=\frac{1}{a^{t}}+\sum_{n=1}^{\infty }\frac{z^{n}}{{\left(n+a\right)}^{t}} \end{array}$$

(*a* ∈ *ℂ*/*ℤ*_0_^−^, *ℤ*_0_^−^ = {0, −1, −2,…}; *t* ∈ *ℂ* when *z* ∈ *U* = U* ⊂ {0}; *ℜ*(*t*) > 1 when *z* ∈ ∂*U*).

Important special cases of the function Φ(*z*, *t*, *a*) include, for example, the Riemann zeta function *ζ*(*t*) = Φ(1, *t*, 1), the Hurwitz zeta function *ζ*(*t*, *a*) = Φ(1, *t*, *a*), the Lerch zeta function *l*_*t*_(*ζ*) = Φ(exp⁡^2*πiξ*^, *t*, 1), (*ξ* ∈ ℝ, *ℜ*(*t*) > 1), and the polylogarithm *L*_*t*_^*i*^(*z*) = *z*Φ(*z*, *t*, *a*). Recent results on Φ(*z*, *t*, *a*) can be found in the expositions [5, 6]. By making use of the following normalized function we define

(9) Gt,a(z)=(1+a)t[Φ(z,t,a)−at+1z(1+a)t]=1z+∑n=1∞(1+an+a)tzn, (z∈U∗).

Corresponding to the functions *G*_*t*,*a*_(*z*) and using the Hadamard product for *f*(*z*) ∈ Σ, we define a new linear operator *L*_*t*,*a*_(*α*, *β*) on Σ by the following series:

(10) Lat(α,β)f(z)=ϕ(α,β;z)∗Gt,a(z)=1z+∑n=1∞(α)n+1(β)n+1(1+an+a)tanzn.(z∈U∗).

The meromorphic functions with the generalized hypergeometric functions were considered recently by many others; see, for example, [7–12].

It follows from (10) that

(11) z(Lat(α,β)f(z))′ =α(Lat(α+1,β)f(z))−(α+1)Lat(α,β)f(z).

In order to prove our main results, we recall the following lemma according to Yang [13].

**Lemma 1**

Let *q*(*z*) = 1 + *q*_*n*_ *z* ^*n*^ + *q*_*n*+1_ *z* ^*n*+1^ + ⋯ be analytic functions in *U* = U* ∪ {0} with *q*(*z*) ≠ 0 for *z* ∈ *U*. If

(12) $$\begin{array}{c} ℜ\left\{1+a\frac{zq^{\prime }\left(z\right)}{q^{2}\left(z\right)}\right\}<M,\;\left(z\in U\right), \end{array}$$

where *a* > 0, and

(13) $$\begin{array}{c} 1<M\leq \frac{n\;a}{2\log2}, \end{array}$$

then

(14) $$\begin{array}{c} ℜ\left\{\frac{1}{q\left(z\right)}\right\}>1-\frac{2\left(M-1\right)}{na}\log2,\;\left(z\in U\right). \end{array}$$

The bound in (14) is the best possible.

## 3. Main Results

We begin with the following theorem.

**Theorem 2**

Let *α* + 1 > 0, *L*_*a*_^*t*^(*α*, *β*)*f*(*z*)/*L*_*a*_^*t*^(*α* + 1, *β*)*f*(*z*) ≠ 0 for *z* ∈ U* and suppose that

(15) ℜ{1+Lat(α+1,β)f(z)(α+1)Lat(α,β)f(z)(1+αLat(α+1,β)f(z)Lat(α,β)f(z))  −Lat(α+2,β)f(z)Lat(α,β)f(z)}<M,

where

(16) $$\begin{array}{c} \;1<M\leq \frac{n}{2\left(\alpha +1\right)\log2}. \end{array}$$

Then

(17) ℜ{Lat(α+1,β)f(z)Lat(α,β)f(z)} >1−2(α+1)(M−1)nlog⁡2, (z∈U∗).

The bound in (17) is the best possible.

**Proof**

Define the function *q*(*z*) by

(18) $$\begin{array}{c} q\left(z\right)=\frac{L_{a}^{t}\left(\alpha ,\beta \right)f\left(z\right)}{L_{a}^{t}\left(\alpha +1,\beta \right)f\left(z\right)}. \end{array}$$

Then, clearly *q*(*z*) = 1 + *q*_*n*_ *z* ^*n*^ + *q*_*n*+1_ *z* ^*n*+1^ + ⋯ analytic function in U* with *q*(*z*) ≠ 0 for *z* ∈ U*. It follows from (18) and (11) that

(19) $$\begin{array}{c} \frac{zq^{\prime }\left(z\right)}{q\left(z\right)}=\frac{z{\left(L_{a}^{t}\left(\alpha ,\beta \right)f\left(z\right)\right)}^{\prime }}{L_{a}^{t}\left(\alpha ,\beta \right)f\left(z\right)}-\frac{z{\left(L_{a}^{t}\left(\alpha +1,\beta \right)f\left(z\right)\right)}^{\prime }}{L_{p}^{*}\left(a+1,c\right)f\left(z\right)} \end{array}$$

by making use of the familiar identity (11) in (19), we obtain

(20) $$\begin{array}{c} \frac{L_{a}^{t}\left(\alpha +2,\beta \right)f\left(z\right)}{L_{a}^{t}\left(\alpha +1,\beta \right)f\left(z\right)}=\frac{1}{\alpha +1}+\frac{1}{\left(\alpha +1\right)q\left(z\right)}-\frac{zq^{\prime }\left(z\right)}{\left(\alpha +1\right)q\left(z\right)} \end{array}$$

or, equivalent,

(21) 1+1(α+1)zq′(z)q2(z) =1+Lat(α+1,β)f(z)(α+1)Lat(α,β)f(z)(1+αLat(α+1,β)f(z)Lat(α,β)f(z))  −Lat(α+2,β)f(z)Lat(α+1,β)f(z).

Applying Lemma 1, with *a* = 1/(1 + *α*), we get the required result.

Letting *α* = *β* = 1 in Theorem 2, we have the following.

**Corollary 3**

Let *G*_*t*,*a*_(*z*)/*z*(*G*_*t*,*a*_(*z*))′ ≠ 0 for *z* ∈ U* and suppose that

(22) ℜ{1+z(Gt,a(z))′2Gt,a(z)(1+z(Gt,a(z))′Gt,a(z))  −z(Gt,a(z))′+(1/2)(Gt,a(z))′′Gt,a(z)}<M,

where

(23) $$\begin{array}{c} 1<M\leq \frac{n}{4\log2}. \end{array}$$

Then

(24) $$\begin{array}{c} ℜ\left\{\frac{z{\left(G_{t,a}\left(z\right)\right)}^{\prime }}{G_{t,a}\left(z\right)}\right\}>1-\frac{4\left(M-1\right)}{n}\log2,\;\left(z\in U^{*}\right). \end{array}$$

The bound in (24) is the best possible.

Letting *M* = 1 + *n*/4log⁡2 in Corollary 3, we have the following.

**Corollary 4**

Let *G*_*t*,*a*_(*z*)/*z*(*G*_*t*,*a*_(*z*))′ ≠ 0 and *t* = 0 for *z* ∈ U* and suppose that

(25) ℜ{1+zf′(z)2f(z)(1+zf′(z)f(z))−zf′(z)+(1/2)z2f′′(z)f(z)} <1+n4log⁡2.

Then *f*(*z*) is starlike in U*.

**Theorem 5**

Let *δ*(*α* + 1) > 0, *zL*_*a*_^*t*^(*α* + 1, *β*)*f*(*z*) ≠ 0 for *z* ∈ U* and suppose that

(26) $$\begin{array}{c} ℜ\left\{{\left(zL_{a}^{t}\left(\alpha +1,\beta \right)f\left(z\right)\right)}^{\delta }\left(\frac{L_{a}^{t}\left(\alpha +2,\beta \right)f\left(z\right)}{L_{a}^{t}\left(\alpha +1,\beta \right)f\left(z\right)}\right)\right\}<M, \end{array}$$

where

(27) $$\begin{array}{c} 1<M\leq \frac{n}{2\delta \left(\alpha +1\right)\log2}. \end{array}$$

Then

(28) ℜ(zLat(α+1,β)f(z))δ >1−2δ(α+1)(M−1)nlog⁡2, (z∈U∗).

The bound in (28) is the best possible.

**Proof**

Define the function *q*(*z*) by

(29) $$\begin{array}{c} q\left(z\right)={\left(zL_{a}^{t}\left(\alpha +1,\beta \right)f\left(z\right)\right)}^{\delta }. \end{array}$$

Then, clearly *q*(*z*) = 1 + *q*_*n*_ *z* ^*n*^ + *q*_*n*+1_ *z* ^*n*+1^ + ⋯ analytic function in U* with *q*(*z*) ≠ 0 for *z* ∈ U*. It follows from (29) that

(30) $$\begin{array}{c} \frac{zq^{\prime }\left(z\right)}{\delta q\left(z\right)}=\frac{z{\left(L_{a}^{t}\left(\alpha +1,\beta \right)f\left(z\right)\right)}^{\prime }}{L_{a}^{t}\left(\alpha +1,\beta \right)f\left(z\right)}-1. \end{array}$$

by making use of the familiar identity (11) in (30), we get

(31) $$\begin{array}{c} \frac{L_{a}^{t}\left(\alpha +2,\beta \right)f\left(z\right)}{L_{a}^{t}\left(\alpha +1,\beta \right)f\left(z\right)}-1=\frac{1}{\delta \left(\alpha +1\right)}\frac{zq^{\prime }\left(z\right)}{q\left(z\right)}, \end{array}$$

or, equivalent

(32) 1+1δ(α+1)zq′(z)q2(z) =(zLat(α+1,β)f(z))δ(Lat(α+2,β)f(z)Lat(α+1,β)f(z)).

Applying Lemma 1, with *a* = 1/(1 + *α*), we get the required result.

Letting *α* = *β* = 1 in Theorem 5, we have

**Corollary 6**

Let *δ* > 0, *G*_*t*,*a*_(*z*) ≠ 0 for *z* ∈ U* and suppose that

(33) $$\begin{array}{c} ℜ\left\{{\left(zG_{t,a}(z)\right)}^{\delta }\left(\frac{z{\left(G_{t,a}(z)\right)}^{\prime }+\left(1/2\right){\left(G_{t,a}(z)\right)}^{\prime \prime }}{G_{t,a}\left(z\right)}\right)\right\}<M, \end{array}$$

where

(34) $$\begin{array}{c} 1<M\leq \frac{n}{4\delta \log2}. \end{array}$$

Then

(35) $$\begin{array}{c} ℜ{\left(zG_{t,a}(z)\right)}^{\delta }>1-\frac{4\delta \left(M-1\right)}{n}\log2,\;\left(z\in U^{*}\right). \end{array}$$

The bound in (35) is the best possible.

Letting *δ* = 1, *M* = 1 + *n*/8log⁡2, and *t* = 0 in Corollary 6, we have the following.

**Corollary 7**

Let *f*′(*z*) ≠ 0 for *z* ∈ U* and suppose that

(36) $$\begin{array}{c} ℜ\left\{zf{\left(z\right)}^{\prime }\left(1+\frac{zf^{\prime \prime }\left(z\right)}{2f^{\prime }\left(z\right)}\right)\right\}<1+\frac{n}{8\log2}. \end{array}$$

Then

(37) $$\begin{array}{c} ℜ\left\{zf{\left(z\right)}^{\prime }\right\}>0,\;\left(z\in U^{*}\right). \end{array}$$

The result is sharp.

**Theorem 8**

Let *ξ* > 0, *z*(*L*_*a*_^*t*^(*α*+1,*β*)*f*(*z*))′/*L*_*a*_^*t*^(*α*, *β*)*f*(*z*) ≠ 0 for *z* ∈ U* and suppose that

(38) ℜ{1+(Lat(α,β)f(z)Lat(α+1,β)f(z))ξ  ×((α+1)Lat(α+2,β)f(z)Lat(α+1,β)f(z))  −α(Lat(α,β)f(z)Lat(α+1,β)f(z))ξ−1}<M,

where

(39) $$\begin{array}{c} 1<M\leq 1+\frac{n}{2\xi \log2}. \end{array}$$

Then

(40) ℜ(Lat(α,β)f(z)Lat(α+1,β)f(z))ξ >1−2ξ(M−1)nlog⁡2 (z∈U∗).

The bound in (40) is the best possible.

**Proof**

Define the function *q*(*z*) by

(41) $$\begin{array}{c} q\left(z\right)={\left(\frac{L_{a}^{t}\left(\alpha +1,\beta \right)f\left(z\right)}{L_{a}^{t}\left(\alpha ,\beta \right)f\left(z\right)}\right)}^{\xi }. \end{array}$$

Then, clearly *q*(*z*) = 1 + *q*_*n*_ *z* ^*n*^ + *q*_*n*+1_ *z* ^*n*+1^ + ⋯ analytic function in U* with *q*(*z*) ≠ 0 for *z* ∈ U*. Also by a simple computation and by making use of the familiar identity (11), we find from (41) that

(42) 1+1ξzq′(z)q2(z)=1+(Lat(α,β)f(z)Lat(α+1,β)f(z))ξ×((α+1)Lat(α+2,β)f(z)Lat(α+1,β)f(z)  −α(Lat(α,β)f(z)Lat(α+1,β)f(z))ξ−1).

Applying Lemma 1, with *a* = 1/*ξ*, we get the required result.

Letting *α* = *β* = 1 in Theorem 8, we have the following.

**Corollary 9**

Let *ξ* > 0, *z*(*G*_*t*,*a*_(*z*))′/*G*_*t*,*a*_(*z*) ≠ 0 for *z* ∈ U* and suppose that

(43) ℜ{1+(Gt,a(z)z(Gt,a(z))′)ξ  ×(1+z(Gt,a(z))′′(Gt,a(z))−(Gt,a(z)z(Gt,a(z))′)ξ)}<M,

where

(44) $$\begin{array}{c} 1<M\leq 1+\frac{n}{2\xi \log2}. \end{array}$$

Then

(45) $$\begin{array}{c} ℜ{\left(\frac{G_{t,a}(z)}{z{\left(G_{t,a}(z)\right)}^{\prime }}\right)}^{\xi }>1-\frac{2\xi \left(M-1\right)}{n}\log2,\;\left(z\in U\right). \end{array}$$

The bound in (45) is the best possible.

Letting *ξ* = 1, *M* = 1 + *n*/2log⁡2, and *t* = 0 in Corollary 9, we have the following.

**Corollary 10**

Let *zf*′(*z*)/*f*(*z*) ≠ 0 for *z* ∈ U* and suppose that

(46) ℜ{1+(f(z)zf′(z))ξ(1+zf′′(z)f′(z)−(f(z)zf′(z))ξ)} <1+n2log⁡2.

Then

(47) $$\begin{array}{c} ℜ\left(\frac{f\left(z\right)}{zf^{\prime }\left(z\right)}\right)>0,\;\left(z\in U^{*}\right). \end{array}$$

The result is sharp.
